# Altered Energetics of Exercise Explain Risk of Rhabdomyolysis in Very Long-Chain Acyl-CoA Dehydrogenase Deficiency

**DOI:** 10.1371/journal.pone.0147818

**Published:** 2016-02-16

**Authors:** E. F. Diekman, G. Visser, J. P. J. Schmitz, R. A. J. Nievelstein, M. de Sain-van der Velden, M. Wardrop, W. L. Van der Pol, S. M. Houten, N. A. W. van Riel, T. Takken, J. A. L. Jeneson

**Affiliations:** 1 Department of Metabolic Diseases, Wilhelmina Children’s Hospital, University Medical Center Utrecht, Utrecht, the Netherlands; 2 Laboratory of Genetic Metabolic Diseases, Departments of Clinical Chemistry and Pediatrics, Emma Children’s Hospital, Academic Medical Center, University of Amsterdam, Amsterdam, the Netherlands; 3 Computational Biology Group, Department of Biomedical Technology, Eindhoven University of Technology, Eindhoven, the Netherlands; 4 Systems Bioinformatics, Department of Life and Earth Sciences, Vrije Universiteit, Amsterdam, the Netherlands; 5 Department of Radiology, University Medical Center Utrecht, Utrecht, the Netherlands; 6 Child Development & Exercise Center, Wilhelmina Children’s Hospital, University Medical Center Utrecht, Utrecht, the Netherlands; 7 Rudolf Magnus Institute of Neuroscience, Spieren voor Spieren Children’s Center, Department of Neurology and Neurosurgery, University Medical Center Utrecht, Utrecht, the Netherlands; 8 Laboratory of Liver, Digestive and Metabolic Diseases, Department of Pediatrics, University Medical Center Groningen, Groningen, the Netherlands; Mayo Clinic, UNITED STATES

## Abstract

Rhabdomyolysis is common in very long-chain acyl-CoA dehydrogenase deficiency (VLCADD) and other metabolic myopathies, but its pathogenic basis is poorly understood. Here, we show that prolonged bicycling exercise against a standardized moderate workload in VLCADD patients is associated with threefold bigger changes in phosphocreatine (PCr) and inorganic phosphate (Pi) concentrations in quadriceps muscle and twofold lower changes in plasma acetyl-carnitine levels than in healthy subjects. This result is consistent with the hypothesis that muscle ATP homeostasis during exercise is compromised in VLCADD. However, the measured rates of PCr and Pi recovery post-exercise showed that the mitochondrial capacity for ATP synthesis in VLCADD muscle was normal. Mathematical modeling of oxidative ATP metabolism in muscle composed of three different fiber types indicated that the observed altered energy balance during submaximal exercise in VLCADD patients may be explained by a slow-to-fast shift in quadriceps fiber-type composition corresponding to 30% of the slow-twitch fiber-type pool in healthy quadriceps muscle. This study demonstrates for the first time that quadriceps energy balance during exercise in VLCADD patients is altered but not because of failing mitochondrial function. Our findings provide new clues to understanding the risk of rhabdomyolysis following exercise in human VLCADD.

## Introduction

The mitochondrial enzyme very-long chain Acyl-CoA dehydrogenase (VLCAD, OMIM 201475) is the first enzyme in the fatty acid oxidation cycle and, as such, a key enzyme in this pathway for mitochondrial energy transduction from fatty acids [1,2]. The clinical presentation of patients with VLCAD deficiency (VLCADD) varies from death in early childhood, associated with fasting intolerance and failing glucose homeostasis [1,3], to exercise intolerance with episodic rhabdomyolysis and myalgia in (early-)childhood and adult life, to asymptomatic individuals [3–5]. In the past decade VLCADD has been included in newborn screening programs all over the world [6]. Treatment consists mainly of dietary advices aimed at prevention of catabolism [7]. However, patients still suffer from exercise intolerance and myalgia, with risk of episodic rhabdomyolysis [8].

Rhabdomyolysis is a grave complication in VLCADD that may lead to kidney damage and even renal failure [4] and can be triggered by prolonged or intense exercise, prolonged fasting and fever or illness. Post-exercise rhabdomyolysis has also been described in other metabolic myopathies including glycogen storage disease [9,10] and statin-induced myopathy (18). The pathophysiological basis of rhabdomyolysis following exercise in these diseases is, however, incompletely understood. A first and longstanding hypothesis invokes myocellular ATP depletion during exercise followed by irreversible myocellular calcium overload [9,10]. In VLCADD, failing myocellular ATP homeostasis during prolonged or intense exercise may result from inhibition of mitochondrial respiration by accumulated incompletely oxidized long-chain fatty acids. Evidence for such an inhibitory mechanism has been found *in vitro* [11]. However, a case study of VLCADD in a patient with a history of rhabdomyolysis following prolonged exercise reported normal mitochondrial ATP synthetic function *in vivo* [12]. Any consistent association between impaired mitochondrial oxidative metabolism and rhabdomyolysis is, in fact, lacking. For example, rhabdomyolysis is a common complication in mitochondrial fatty acid oxidation defects [13–16], but rare in primary defects in mitochondrial oxidative phosphorylation [17,18]. An alternative hypothesis specifically for defects in fat oxidation invokes direct damage to the myocellular membrane by high concentrations of incompletely oxidized acyl-carnitines that accumulate during prolonged or intense exercise [18]. Supportive evidence for this hypothesis has likewise been found *in vitro* [19–21]. However, the absence of any severe cardiomyopathic phenotype in adult VLCADD patients (thesis E.F. Diekman 2015) may argue against the relevance of such a pathophysiological mechanism in vivo.

Here, we investigated if muscle ATP homeostasis during prolonged stationary exercise at a standardized workload corresponding to maximal fat oxidation (FATMAX) in healthy subjects is compromised in VLCADD patients. Five VLCADD patients and five healthy controls performed a maximal cardiopulmonary exercise test (CPET) to determine the workload corresponding to their individual maximal rate of fat oxidation. During a second visit to the hospital, subjects then performed 45 minutes of bicycling exercise at their individual FATMAX workload. Blood samples were drawn at various timepoints during the protocol for selective metabolite profiling. The last five minutes of the exercise task were performed inside a clinical MRI scanner for continuous in vivo phosphorus nuclear magnetic resonance spectroscopy (^31^P MRS) recordings of the concentrations of ATP, phosphocreatine (PCr) and inorganic phosphate (Pi) as well as intramuscular pH in the quadriceps muscle during bicycling exercise at FATMAX and subsequent recovery. The time course data of post-exercise PCr and Pi recovery to basal levels were applied to a linear model of muscle respiration[22] to determine the mitochondrial capacity for ATP synthesis of quadriceps muscle after 45 min of exercise at FATMAX. The steady-state quadriceps concentrations of ATP, PCr and Pi during FATMAX exercise were applied to a computational model of oxidative ATP metabolism in a skeletal muscle composed of slow-twitch oxidative (SO), fast-twitch oxidative glycolytic (FOG) and fast-twitch glycolytic (FG), respectively, to identify combinations of muscle fiber type composition and motor unit recruitment scenarios in healthy subjects and VLCADD patients that were consistent with the ^31^P MRS results. Our results show that prolonged bicycle exercise at FATMAX workload is associated with threefold larger changes in quadriceps PCr and Pi concentration and twofold lower changes in acetyl-carnitine blood levels in VLCADD patients compared to healthy subjects. Yet the rate of PCr and Pi recovery post-exercise in VLCADD patients was normal. Model simulations showed that the observations in VLCADD patients were best explained by a slow-to-fast shift in quadriceps fiber-type composition corresponding to 30% of the slow-twitch fiber-type pool.

## Methods

### Subjects, design and study selection

Five patients with VLCADD (Tables 1 and 2) and five age-matched healthy controls were recruited. Patients with myopathic exacerbation at time of experiment, cardiomyopathy/arrhythmia, epilepsy or pregnancy were excluded from the study, as well as the presence of conventional contra-indications for ^31^P/^1^H MRS (MRI) measurements. The study consisted of two separate exercise test-sessions separated by at least two weeks. First, subjects performed a standard cardio-pulmonary exercise test (CPET) on an upright bicycle ergometer. From the results of this test, the workload corresponding to maximal bodily fat oxidation (FATMAX) was determined for each subject. Two or more weeks later, subjects performed 45 minutes of bicycling exercise at their individual FATMAX workload, of which the final five minutes were performed supine inside the MR scanner using a MR-compatible bicycle ergometer [23]. Blood samples were taken at three timepoints: before, 5 and 180 minutes after the FATMAX exercise test, respectively. The study was approved by the medical ethics committee of the University Medical Centre Utrecht (METC 12-211/K). All patients provided written informed consent for participation in this study.

**Table 1:** Patient characteristics and plasma concentration of several metabolites in resting conditions. PID = Patient Identification number; y = year; M = male/F = female; BMI = body mass index; CK = creatine kinase. Contr. = controls Mean ± SEM is reported.

PID	Age (y)	Sex	Height (cm)	Weight (kg)	BMI	C14:1-carnitine (μmol/L)	Basal CK	# of hospital admissions	Symptoms	Activities in daily life
1	13	M	171	53	18.1	1.2	1553	6	muscle pain	in school; abstains from any voluntary activity
2	17	F	158	79	31.7	1.0	36	11	muscle pain, fatigue	in school; abstains from any voluntary activity
3	32	M	175	74	24.2	8.9	1253	2	muscle pain, fatigue	heavy manual labor; recreational sports (soccer)
4	41	M	187	87	24.9	1.1	156	2	muscle pain, fatigue	not able to work; abstains from any voluntary activity
5	37	M	186	87	25.2	1.8	83	1	asymptomatic	College graduate office worker; recreational outdoor sports
Contr.	26 ± 4	1F, 4M	179±6	71±7	21.8 ± 1.3	0.04 ± 0.1	113±20			

**Table 2:** Mutation analysis of patients.

PID	cDNA substitutions	Predicted amino-acid change
1	homozyg. c.104delC	p.Pro35LeufsX26	
2	homozyg. c.1141-43delGAG	p.Glu381del	
3	homozyg. c.1406G>A	p.Arg469Gln	
4	c.520G>A; c.833_835delAAG	p.Val174Met; p.Lys278del	
5	c.848T>C; c.1444_1448delAAGGA and 1509_1514delAGAGG	p.Val283Ala; p.Lys482AlafsX78 and p.Glu504_Ala505del	

### Dietary standardization

Patients and healthy controls were asked to keep a food record the three days preceding the test day. A light meal was allowed before the test. An extensive dietary analysis based on the three-day food diary and a subsequent interview by a nutritionist, was performed in all patients prior to the second exercise test (NEVO-table 2011 (Dutch Food Composition Table), RIVM/Voedingscentrum, Den Haag 2011).

### Exercise testing

#### I. Cardio-pulmonary exercise test (CPET)

Maximal exercise capacity was measured during the baseline graded CPET[24]. The participant performed exercise on a standard upright bicycle ergometer under increasing load until exhaustion (duration +/- 10min). By using the respiratory gas exchange parameters, the intensity of maximal fat oxidation (FATMAX) could be determined [25]. Subjects sat placed on an upright cycle ergometer and fitted with a 12-lead ECG, a pulse oximeter on the index finger or forehead, an automatic blood pressure cuff, and a small face mask attached to a breath-by-breath gas analysis system. Subjects performed a pulmonary function test at rest to determine the forced expiratory volume in 1 sec (FEV_1_) and forced vital capacity (FVC). After this measurement, subjects began the test with 5 minutes of resting measurements while sitting on the cycle ergometer. A modified protocol for determining the maximal fat oxidation (FATMAX) as recommended for testing on a cycle ergometer (Lode Corival, Lode BV, Groningen, the Netherlands) was used[26]. The subjects were instructed to cycle at an increasing load of 35 watts every 3 minutes, starting with 0 watts and ending at voluntary exhaustion. During this time several measurements were taken: a full electrocardiogram (ECG; Cardioperfect, Accuramed BVBA, Lummen, Belgium), oxygen (O_2_) saturation (Masimo, Rad 8, Masimo BV, Tilburg, the Netherlands), heart rate (HR), blood pressure (BP) (Suntech Tango, Suntech Med, Morrisville, NC, USA), minute ventilation (VE), oxygen uptake (VO_2_), carbon dioxide output (VCO_2_), respiratory exchange ratio (RER), workload (W), using a calibrated metabolic cart (ZAN 600, Accuramed BVBA, Lummen, Belgium). Subjects were asked to keep their cadence per minute between 60–80, and were given verbal encouragement to continue if they fell below that range. Immediately after exhaustion, the load was decreased to 0 watts and subjects were asked to cool down for 5 minutes. FATMAX workload was calculated according to the formula proposed by Péronnet and Massicotte for calculating fat oxidation, as it is both accurate in estimating fat oxidation and does not require an estimate of protein degradation during exercise (Eq (1);[27]:

$$Fat\;oxidation=1.695∙{VO}_{2}-1.701∙{VCO}_{2}$$ eqn (1)

where fat oxidation is in mg·min^-1^, and VO_2_ and VCO_2_ are both In mL·min^-1^. Workload, the independent variable for the FATMAX curve, was taken in terms of HR, Watt, and percentage of VO_2peak_. The final 60 seconds of exercise for each workload interval were averaged to achieve steady-state values. Each set of steady-state FATMAX values were fit along a second order polynomial curve.

#### II. Endurance exercise test

Subjects were asked to exercise at their individual FATMAX workload for a total of 45 minutes. Of these, 40 minutes were performed on an upright standard bicycle ergometer (Lode, Groningen, the Netherlands) in a room adjacent to the MRI scanner. Next, subjects were moved to the MR scanner and performed the final five minutes of exercise task in supine position inside the MR scanner at a workload equivalent to FATMAX. Hereto, the braking force on the MR-compatible bicycle ergometer was gravimetrically adjusted to the appropriate amount using 30 N as empirical reference for maximal sustainable braking force in healthy subjects (data not shown).

### ^31^P-Magnetic Resonance Spectroscopy

Subjects were positioned feet-forward in a supine position in a 1.5T Philips MR Achieva scanner (Philips Healthcare, Best, the Netherlands). The upper body was supported by a wedge-shaped cushion to facilitate supine bicycling. A 6-cm diameter single-turn ^31^P surface coil supplied by the manufacturer was fastened over the m. vastus lateralis of the right leg. Subjects then performed a short bout of unloaded bicycling supervised by an on-site coach to familiarize them with the supine exercise task. The desired pedalling frequency (target setting: 75 rpm) was set by a metronome audible over the in-magnet speaker system.[24] This training exercise bout was typically performed within five min after the conclusion of the upright FATMAX bicycling test. Next, a series of scout MR images was acquired to evaluate correct positioning of the subject and the coil and image-based shimming was performed. ^31^P NMR spectra were acquired from the m. vastus lateralis at rest, during exercise and recovery using an adiabatic half-passage excitation pulse as described elsewhere [23]. First, a resting spectrum was acquired under fully-relaxed conditions (repetition time (TR) 20 s). During exercise and recovery, four and two free induction decays (FIDs), respectively, were acquired with a TR of 3s and averaged, yielding time resolution of 12 and 6 seconds, respectively, in each dynamic phase. [24]

### ^31^P-MRS data processing

FIDs were processed and analyzed in the time domain using the AMARES algorithm in the public jMRUI software environment (version 3.0) as described elsewhere[23]. Kramer-Rao bounds of the AMARES Lorentzian model fitting were used as statistical information on accuracy of peak area estimation. Absolute PCr and Pi concentrations were calculated after correction for signal saturation assuming total adenylate nucleotide and creatine pool sizes of 8.2 and 42.7 mM, respectively, as previously described[28]. Intramuscular pH was determined from the resonance frequency of Pi using standard methods[28]. Steady-state PCr and Pi concentrations during exercise were determined from summed FIDs acquired 60s after onset of bicycling. Datasets were analysed in a blinded fashion.

### Computational Modeling

This study employed a multi-fiber computational model of human muscle oxidative ATP metabolism to analyze the in vivo ^31^P MRS data obtained during exercise. This approach was necessary to address two intrinsic sources of microscopic metabolic heterogeneity in exercising quadriceps muscle. First of all, human skeletal muscles are composed of SO, FOG and FG fiber types organized in motor units of uniform fiber type that vary in properties including size, contractile speed, ATP cost of contraction and relaxation, power-output, capacity for fat and carbohydrate metabolism, adenine nucleotide and creatine pool sizes, mitochondrial density and vascularisation [29]. Secondly, during voluntary exercise motor units are neurally recruited in a stepwise order according to the size principle–i.e., SO < FOG < FG [30]. As a result, any ^31^P NMR spectrum of human muscle engaged in a submaximal voluntary exercise task reflects the average of the particular concentrations of PCr, Pi and ATP and intramuscular pH in SO, FOG and FG fibers, respectively, including active and inactive fibers within the sampled tissue volume. Investigations of metabolic changes in individual fiber types employing dissection of biopsy specimens have previously demonstrated profound differences in PCr content and glycogen depletion between slow and fast fibers[31]. A multi-fiber kinetic model of oxidative ATP metabolism in human skeletal muscle was developed to investigate if, and if so, what magnitude of fiber type composition changes in VLCADD skeletal muscle may contribute to any measured average change in stationary states of energy balance at FATMAX exercise in VLCADD versus healthy muscle. A kinetic model of oxidative ATP metabolism described in detail elsewhere [32] was used as core model in each fiber type submodel. Fiber-type specific submodel parameter values with regard to oxidative ATP metabolism and default healthy quadriceps composition are given in Table A in S1 File; other model parameters were assumed to be uniform across fiber types. The relation between measured average Pi and PCr concentrations in the quadriceps muscle mass sampled by the ^31^P surface coil and fiber-type-specific concentrations is described by (Eq (2))

$$M_{average}=X_{SO}∙M_{SO}+X_{FOG}∙M_{FOG}+X_{FG}∙M_{FG}$$ eqn (2)

where *M*_SO_, *M*_FOG_, *M*_FG_ denote the fiber-type specific concentration of metabolite PCr or Pi according to the model, and *X*_SO_, *X*_FOG_, *X*_FG_ denote the fraction of quadriceps muscle composed of SO, FOG and FG fibers, respectively. The default model parameterization for X_tSO_, X_FOG_, X_FG_ was 0.5, 0.35 and 0.15, respectively [33]. For the simulations, it was assumed that the maximal mechanical output that muscle fibers can sustain without any significant fatigue is associated with a cellular oxygen consumption rate equal to 80% of maximal rate [34,35]. The model was implemented in Matlab 7.5.0 (The Mathworks, Natick, MA, USA). Ordinary differential equations were solved numerically by using ODE15s with absolute and relative tolerances set to 10^−8^.

### Analysis of blood samples

Plasma of blood samples were profiled for glucose, lactate, acylcarnitines and creatine kinase. Plasma was stored at -20°C. Plasma acylcarnitines were measured as described previously [36] using internal standards. Blood for measurement of lactate and pyruvate was treated immediately after withdrawal with an equal volume of 1M perchloric acid and subsequently stored at -20°C. Plasma was stored at -20°C. Plasma glucose and CK were measured using standard enzymatic assays [37]. Blood lactate and pyruvate, were measured using tandem mass spectrometry[38]. All samples were analysed in a blinded fashion.

#### Statistics

Two-tailed Mann Whitney U-tests were used to determine significant differences between control and patients at p <0.05. Data are presented as mean ± standard error of mean (SEM) unless specified otherwise. Time courses of variables were analysed and characterized kinetically using non-linear curve-fitting (Origin 6.0, Caltech Pasadena, US). Whenever possible, statistical information on data accuracy was incorporated in the curve-fitting by means of statistical weighting.

## Results

### Baseline characteristics of the patients

Five VLCADD patients (Table 1) and five healthy subjects were included in the study. Diagnosis of VLCADD was confirmed by mutation analysis of the ACADVL gene (Table 2). Four patients had exercise intolerance, myalgia and fatigue while one patient (PID#5) was asymptomatic. Three patients had a residual VLCAD enzymatic activity of ≤5% in fibroblasts (PID#1–3), and in two patients residual VLCAD activity was ≤13% (compared to reference control fibroblasts). The symptomatic patients had been admitted to the hospital at least twice in their lives (range 2–11) (Table 1). Plasma creatine kinase level was increased mildly in two of the patients. C14:1-carnitine was significantly increased in VLCADD patients compared to controls (p = 0.02). Age, height, weight and BMI were comparable between controls and VLCADD patients (Table 1). ^31^P metabolite concentrations and intramuscular pH in resting quadriceps muscle were not different between VLCADD patients and healthy controls (Data not shown). Dietary intake of the last three days before the second test did not differ significantly between controls and VLCADD patients (Table B in S1 File).

### Maximal exercise capacity is reduced in VLCADD patients

**Fig 1:**
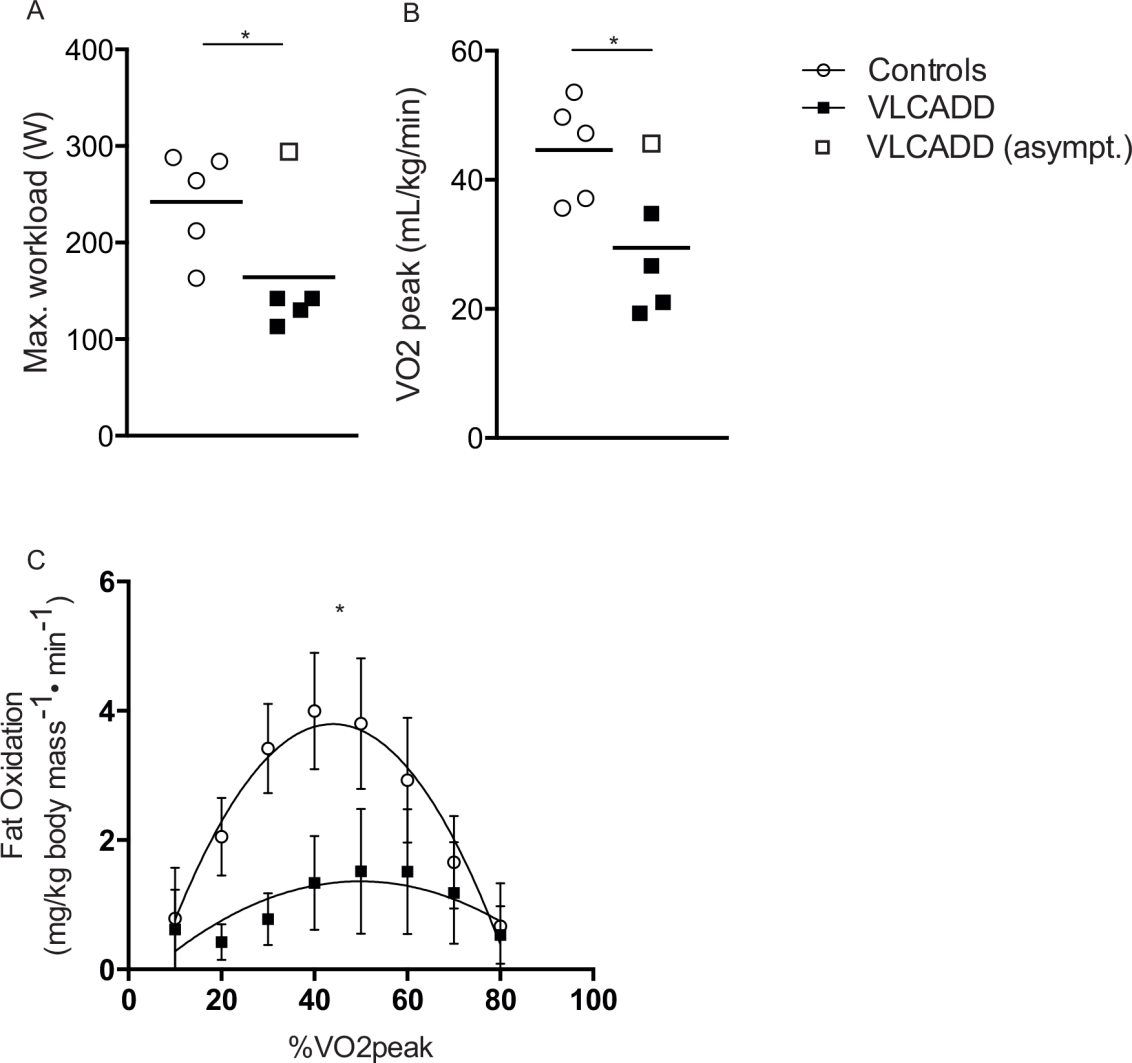
(A) Maximal workload of symptomatic (black squares) and asymptomatic (open squares) VLCADD patients and controls. (B) in symptomatic (black squares) and asymptomatic (open squares) VLCADD patients. (C) Fatty acid oxidation in mg/kg body mass/min in VLCADD patients and controls. Error bars indicate ± SEM. *P<0.05 for polynominal curve.

The maximal sustained workload during CPET in the four symptomatic VLCADD patients was 46 ± 6% reduced compared to healthy controls (Table 3 and Fig 1A). In contrast, the maximal workload attained by the asymptomatic patient was well within the normal range (Table 3). Two patients (PID #2 and #3) had a RER_peak_ comparable to controls, while the other three patients (including the asymptomatic patient) had an elevated RER_peak_ between 0.90 and 1.04. VO_2_peak_ was 55% decreased in the four myopathic patients (Table 3 and Fig 1B). The estimated peak rate of fat oxidation per kg of bodymass was 46% lower in myopathic patients compared to controls (Fig 1C).

**Table 3:** Graded CPET exercise test characteristics of controls and patients. HRpeak = peak heart rate; RER = respiratory exchange ratio. Mean ± SEM is reported.

PID	Max workload (W)	HR _peak_ (beats/min)	Resting RER	VO_2peak_ (mL/kg/min)	Peak ventilation (L/min)	Max Fat oxidation (% of VO_2_peak)	Max Fat oxidation (mg/kg/min)	FATMAX intensity (W)
1	142	185	0.90	34.8	58	30.0	0.85	3
2	142	182	0.84	26.6	60	55.0	4.13	50
3	113	149	0.83	19.3	36	54.5	2.09	32
4	130	175	1.04	21.0	79	29.0	0.0	0
5	294	184	0.94	45.6	143	41.7	2.52	81
Contr.	242±24	189±5	0.83±0.02	45±4	114±11	38±4	3.8±1.0	51±16

To investigate if ATP homeostasis in muscle of VLCADD patients is compromised during prolonged stationary exercise at a normalized workload corresponding to maximal fat oxidation in healthy subjects (FATMAX), subjects reported to the MRI Center two weeks or more after CPET to perform a second exercise test consisting of 45 min of bicycling at their individual FATMAX workload. The first 40 min of the test involved conventional upright bicycling exercise. Immediately afterwards, subjects were transferred to the MRI scanner room and mounted on a MR-compatible bicycle ergometer for in vivo ^31^P MR spectroscopic measurement of intramuscular energy balance and pH in the quadriceps during the remaining five minutes of the exercise test. All subjects were able to complete the test except patient PID#4 who did manage to complete 43 of the requested 45 minutes of exercise at FATMAX.

### Metabolic profiling of blood samples shows abnormal dynamics of plasma acetyl-carnitine levels in exercising VLCADD patients

**Fig 2:**
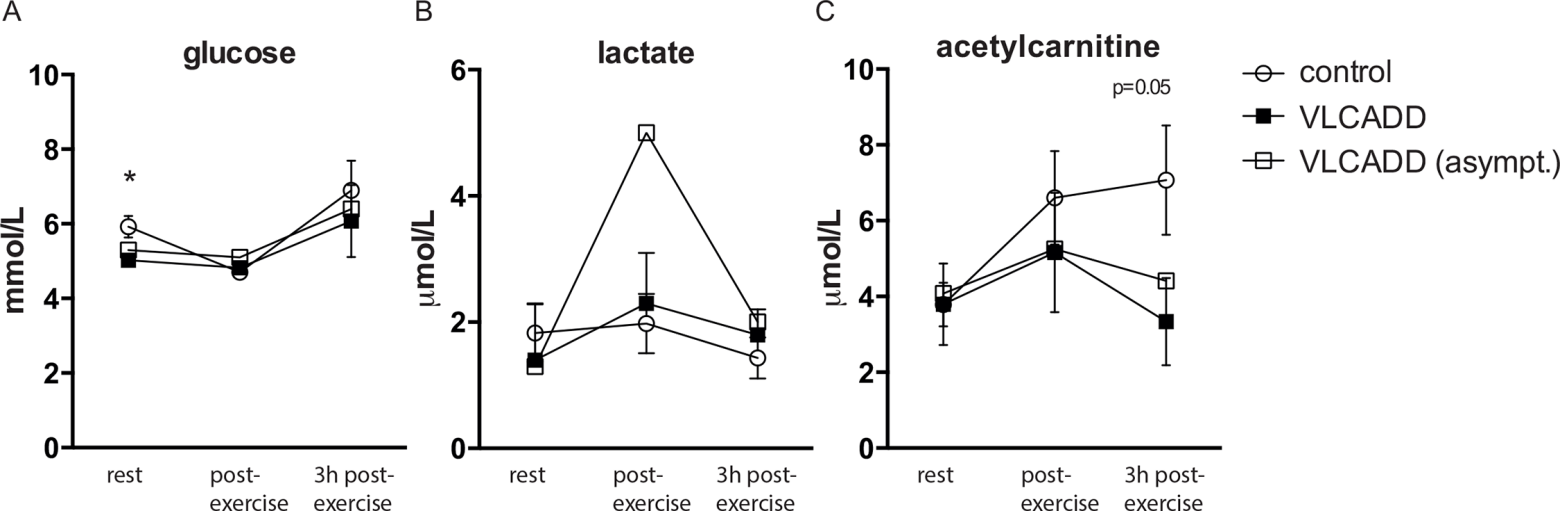
(A) Glucose, (B) lactate and(C) acetylcarnitine at t = 0 (rest), t = 1 (directly after exercise) and t = 2 (3 hours after exercise) in symptomatic VLCADD patients and controls. Error bars indicate mean ± SEM, *P<0.05.

Glucose levels remained unchanged (Fig 2A). Lactate levels were not different between controls and myopathic VLCADD patients (Fig 2B). In the asymptomatic patient lactate levels increased fourfold from 1.3 to 5 mmol/L during exercise at FATMAX (Fig 2B). Basal CK levels were normal (i.e., (<250U/L) in all controls and three VLCADD patients (Table 1). In response to the FATMAX exercise test, CK levels in two myopathic patients slightly increased (PID #2 and #4; 400 and 576 U/L, respectively) but still remained low. Resting plasma C14:1-acyl-carnitine levels were significantly higher in VLCADD patients than healthy controls (Table 1). Plasma acetyl-carnitine (C2-carnitine) levels in resting state were similar between patients and healthy controls (Fig 2C). In the time span between onset of exercise and 3 hours post-exercise, plasma acetyl-carnitine levels in healthy controls nearly doubled compared to resting levels, with the majority of the rise occurring over the 40 min exercise test (Fig 2C). In VLCADD patients, plasma acetyl-carnitine levels remained constant.

### *In vivo* 31P MRS identifies altered quadriceps energy balance in VLCADD during exercise at FATMAX workload

**Fig 3:**
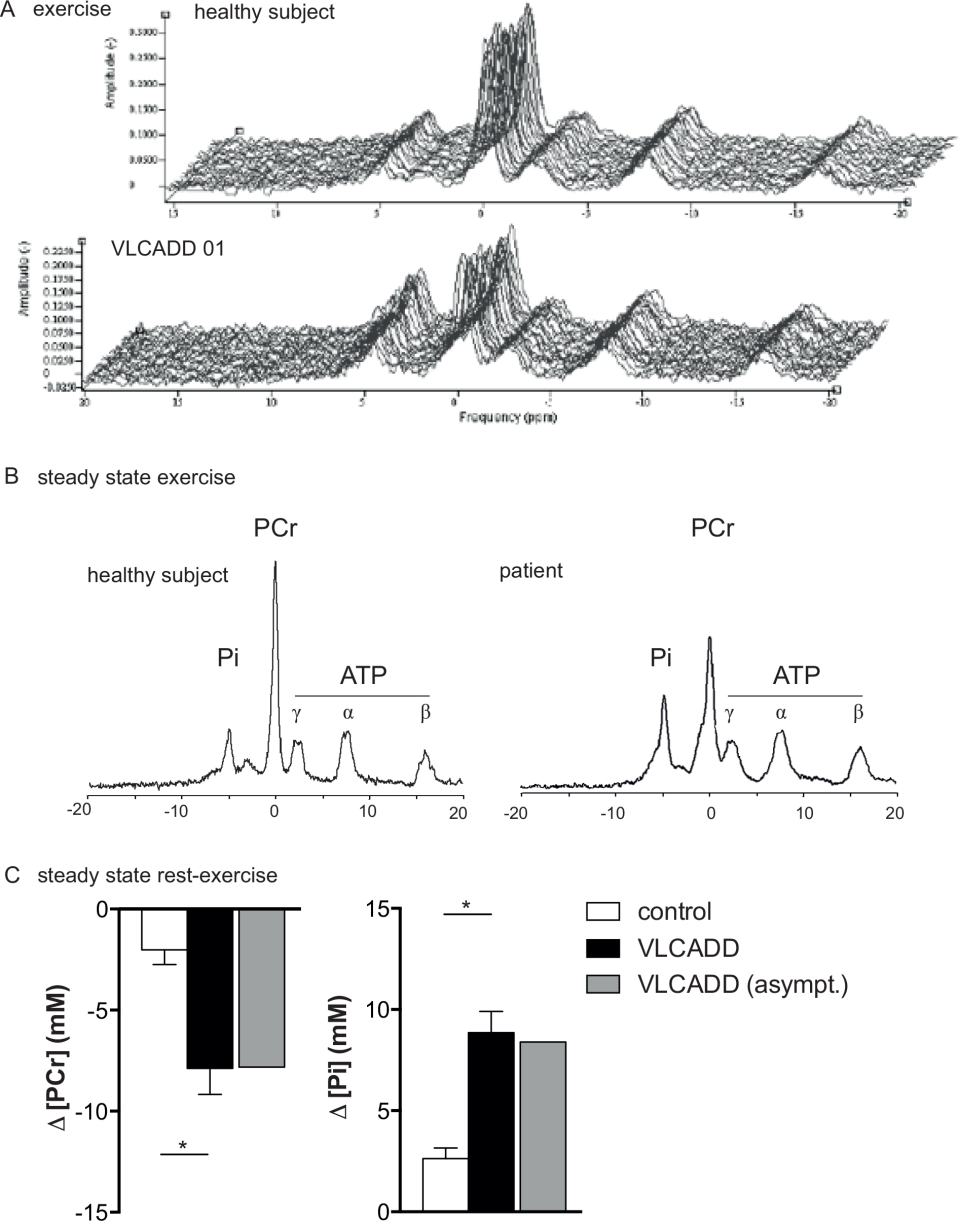
(A) ^31^P NMR spectra acquired from the lateral head of the quadriceps muscle of the right leg of a VLCAD deficient patient versus a healthy control subject during 5 min of bicycling exercise at a workload equivalent to FATMAX in each subject. (B) Each spectrum represents the sum of the FIDs collected after 60 s of exercise. FIDs were apodized in the time domain using a 10-Hz low-pass filter prior to Fourier transform and phasing. Peak assignments: Pi inorganic phosphate, PCr phosphocreatine, ATP adenosine triphosphate (gamma, alpha and beta resonances, respectively). (C) Average change in PCr and Pi during exercise (in mM) in healthy control subjects versus symptomatic and asymptomatic VLCAD deficient patients. Error bars indicate mean ± SEM, *P<0.05.

**Fig 4:**
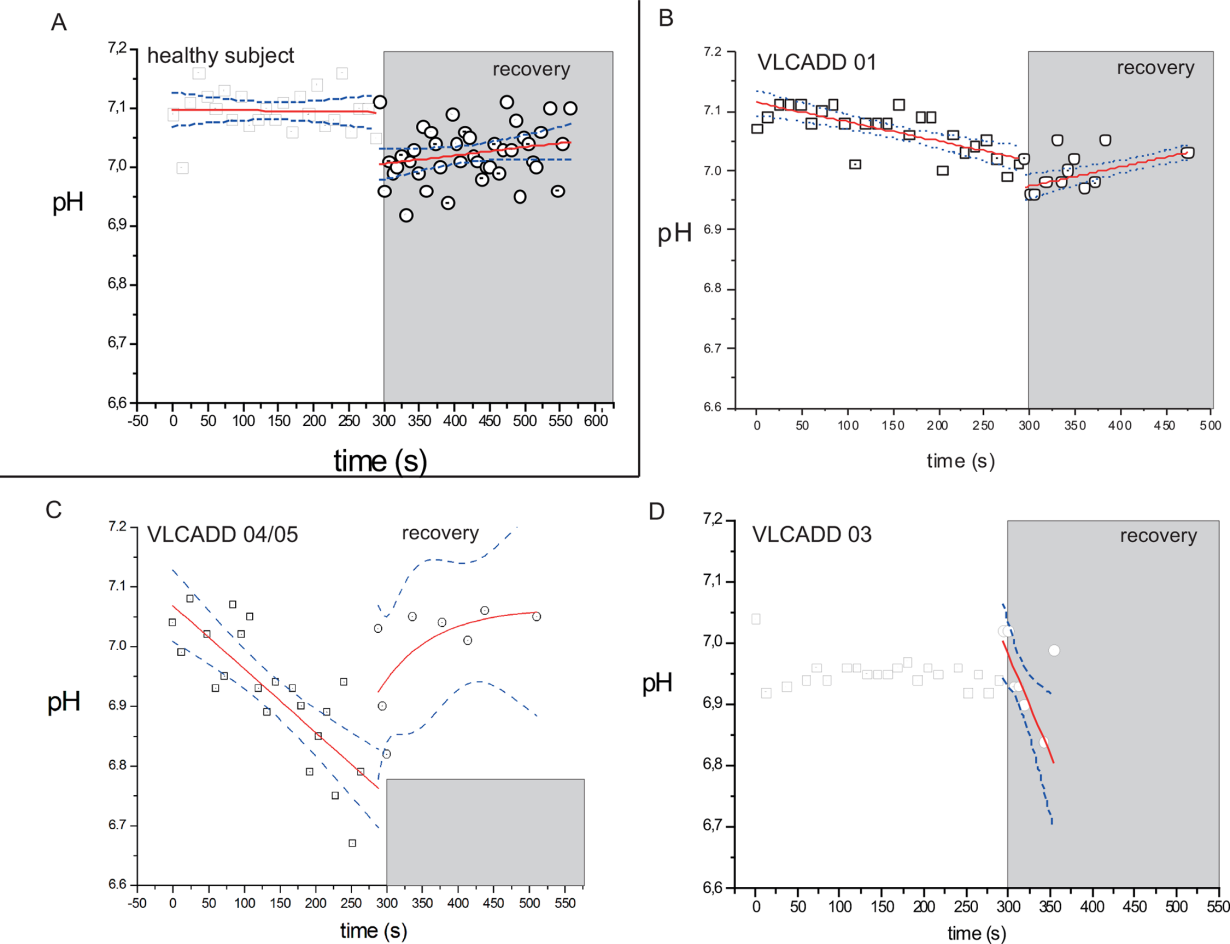
pH dynamics during exercise and first minutes of recovery. The error bars show the variance in the data from AMARES fitting of the MR spectra (see Methods section). (A) Healthy controls. (B-D) VLCADD patients. Solid red lines show the fit of linear or monoexponential functions to the data; blue lines show the 95% confidence interval of the fit.

Fig 3A shows a time series of ^31^P NMR spectra recorded sequentially from the vastus lateralis muscle of a healthy control (upper trace) and a VLCADD patient (lower trace) during supine bicycling exercise at FATMAX workload. Fig 3B shows the corresponding ^31^P NMR spectra of the summed FIDs recorded after 60s into the exercise for these subjects. In all healthy control subjects, only minor changes in steady-state Pi and PCr concentrations from resting values were observed during exercise at FATMAX (ΔPCr: -2.0±0.7 mM; ΔPi: +2.6±1.0 mM (mean + SE) (Fig 3C). In contrast, in both myopathic as well as the asymptomatic VLCADD patients, pronounced changes in quadriceps Pi and PCr concentrations from resting values were observed during exercise at FATMAX (ΔPCr: -7.9 ± 1.0 mM; ΔPi: +8.8 ± 0.8 mM (mean + SE) (Fig 3C). Quadriceps pH during exercise did not fall below 7.0 in any healthy control subject (Fig 4A) but intramuscular pH changes during exercise in VLCADD patients were more heterogeneous. Specifically, in patient PID#1 quadriceps pH did not fall below 7.0 during exercise similar to healthy controls (Fig 4B). In patients PID#02, #04 and #05 intramuscular pH progressively dropped during exercise to values as low as 6.7 (example shown in Fig 4C). In patient #03, intramuscular pH immediately dropped 0.1 units to 6.95 at the onset of exercise and remained stable at this value during the remainder of the exercise (Fig 4D).

### Normal kinetics of post-exercise metabolic recovery rule out impaired mitochondrial ATP synthetic function in VLCADD

**Fig 5:**
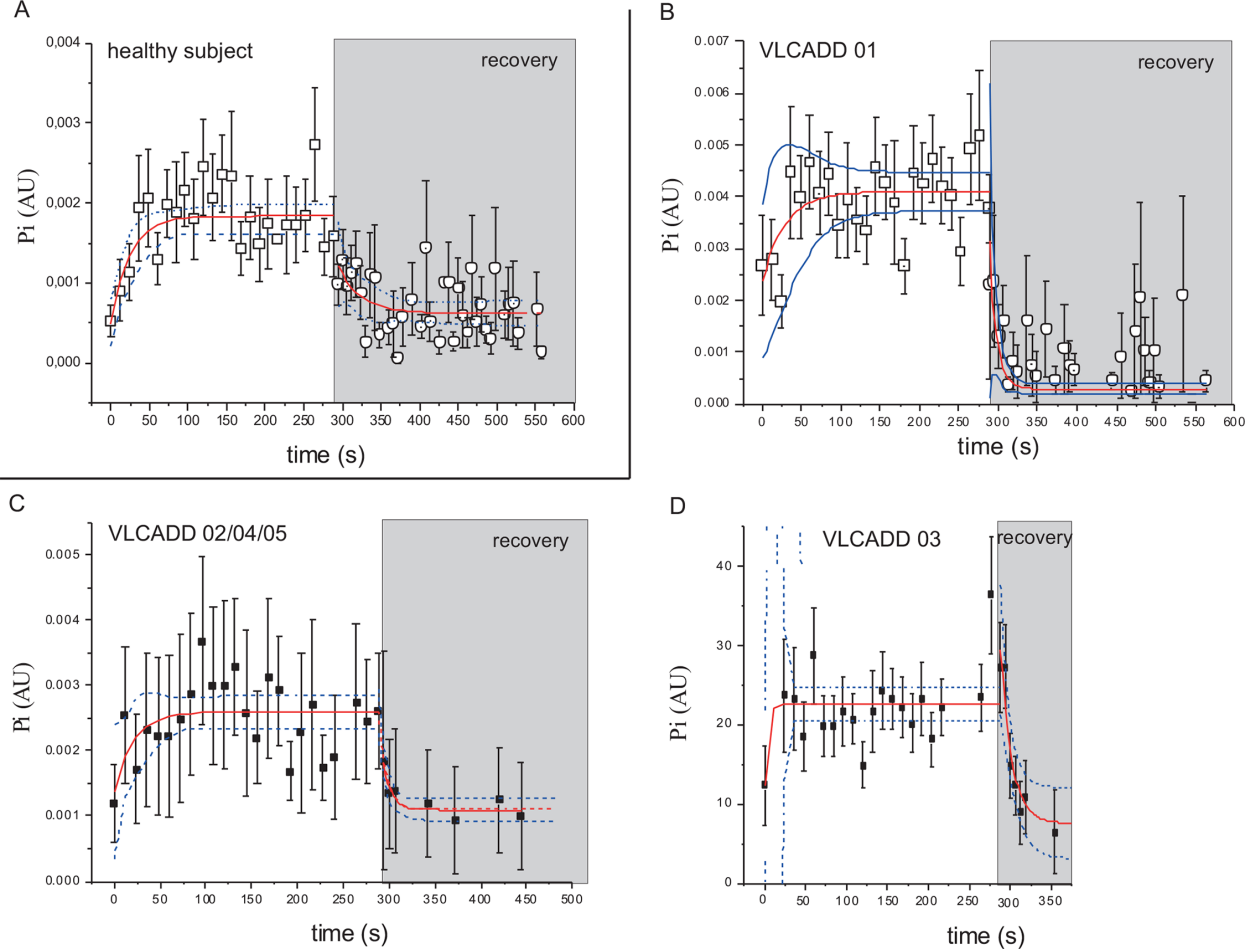
Pi dynamics during exercise and the first minutes of recovery rates. The error bars show the variance in the data from AMARES fitting of the MR spectra (see Methods section). (A) Healthy controls. (B-D) VLCADD patients. Solid red lines show the fit of a monoexponential function to the data; blue lines show the 95% confidence interval of the fit.

To evaluate the capacity of oxidative ATP synthesis in quadriceps muscle of the patients after 45 minutes of exercise at FATMAX, the rate of metabolic recovery to resting state was measured immediately after exercise. In all VLCADD patients, normal kinetics were found for the recovery of PCr and Pi concentrations to basal levels post-exercise compared to typical literature values for human quadriceps muscle (Table 4 and Fig 5; Fig A in S1 File). No major abnormalities were found in the post-exercise dynamics of quadriceps pH in any of the patients compared to healthy controls (Fig 4). The ancillary drop in intramuscular pH at the onset of recovery followed by a slow recovery to resting values typically observed in healthy control subjects (Fig 4A) was likewise observed in patients PID#01 and #03 (Fig 4B and 4D). In the patients PID#02, PID#04 and PID#05 intramuscular pH recovered without any further drop of pH at the onset of recovery (Fig 4C).

**Table 4:** PCr and Pi recovery rates.

PID	tau PCr (s)	tau Pi (s)
1	21 ± 9	12 ± 7
3	ND	12 ± 5
2	16 ± 9	21 ± 13
4	28 ± 13	19 ± 14
5	ND	9 ± 5
Controls	24 ± 5 ^1^	24 ± 5^2^

^1^ value for vastus lateralis muscle of healthy human subjects (22)

^2^ Pi recovery kinetics are similar although not quite identical to PCr recovery[65]. ND = not determined.

### Metabolic model simulations indicate a fast-to-slow shift in fiber type composition of VLCADD muscle explains altered energy balance during exercise

Our findings in the VLCADD patients of altered quadriceps energy balance during exercise but intact capacity for oxidative ATP production after 45 minutes of exercise prompted us to conduct numerical simulations of oxidative ATP metabolism in skeletal muscle composed of three fiber types and compare each outcome to the ^31^P MRS results. In the control subjects, the measured concentrations of PCr and Pi during stationary exercise at FATMAX workload were consistent with recruitment of 70% of the total pool of SO fibers of the quadriceps during the voluntary exercise task (Table 5). If no change was made to the model with respect to quadriceps fiber type composition, the PCr and Pi concentrations measured during exercise in the patients could only be explained by recruitment of 100% of the SO muscle fiber pool (Table 5). Alternatively, if the absolute number of SO fibers in the model was reduced by 30% and replaced by FOG fibers, the ^31^P MRS observations in the patients could be explained by recruitment of 100% of the reduced SO fiber pool plus 35% of the augmented FOG muscle fiber pool (Table 5).

**Table 5:** results of model simulations of motor unit recruitment (in % of total pool size) during voluntary exercise at FATMAX in healthy control subjects and VLCADD patients.

**Scenario I:**	**X**_**type I**_ **= 0.5**	**X**_**type IIA**_ **= 0.35**	**X**_**type IIX**_ **= 0.15**
	SO	FOG	FG
Controls	70%	0%	0%
VLCADD	100%	0%	0%
**Scenario II:**	**X**_**type I**_ **= 0.35**	**X**_**type IIA**_ **= 0.5**	**X**_**type IIX**_ **= 0.15**
	SO	FOG	FG
VLCADD	100%	35%	0%

X_*i*_: fraction of fiber type *i* in composition of quadriceps muscle; three fiber types; sum of all fractions = 1.0. SO: slow oxidative fiber

## Discussion

In this study, we demonstrate that prolonged stationary bicycling exercise against a standardized moderate workload in VLCADD patients is associated with threefold bigger changes in PCr and Pi concentrations in quadriceps muscle and twofold lower changes in plasma acetyl-carnitine levels than in healthy subjects. This result is consistent with the hypothesis that muscle ATP homeostasis during prolonged exercise in VLCADD is compromised. However, kinetic measurements of the recovery of ATP metabolism to resting state immediately after exercise, showed that the mitochondrial capacity for ATP synthesis in VLCADD quadriceps muscle after 45 min of exercise was not impaired. Mathematical model-based simulations of oxidative ATP metabolism in skeletal muscle composed of SO, FOG and FG fiber types showed that these findings may be explained by a slow-to-fast shift in VLCADD quadriceps fiber type composition.

All five VLCADD patients that participated in this study had highly elevated C14:1-carnitine levels in diagnostic studies in fibroblasts consistent with the molecular defect. Here, plasma acetyl-carnitine (C2-carnitine) levels remained constant over the time span between onset of exercise and 3 hours after exercise in the VLCADD patients whereas in the control subjects these metabolite levels almost doubled. The latter finding is consistent with previous findings in healthy subjects [39]. Acetyl-carnitine concentrations in blood are assumed to reflect concentrations of mitochondrial acetyl-CoAs [39]. As such, the observed absence of any increase of blood acetyl-carnitine concentrations in the patients suggests that mitochondrial acetyl-CoA concentrations did not increase under the test conditions of prolonged exercise at FATMAX. This inability to increase acetyl-CoA, could have caused the inability to provide enough ATP to replenish PCr levels. Blood lactate levels did not increase in response to exercise in neither healthy subjects nor patients, except for patient PID#5. This result was consistent with the intended oxidative nature of the exercise task. Indeed, all subjects were able to complete the task with the exception of patient PID#4 who, however, still managed to complete 43 of the 45 min of exercise.

The main result of the present study was the finding of threefold bigger change in quadriceps PCr and Pi concentrations during stationary exercise at FATMAX in VLCADD patients than in healthy control subjects. This result indicates that homeostasis of the concentrations of the ATP hydrolysis products ADP and Pi is less tight in VLCADD muscle than in healthy muscle. The latter is significant because it is thought that control of these concentrations is critical for proper function of myocellular cation pumps that are involved in fiber relaxation [40–42]. Control of the concentrations of ADP and Pi in muscle resides in the network of myocellular enzymes involved in the ATP hydrolysis-driven fiber contraction and relaxation [42,43]. Therefore, our observation raised the question of which network components and/or their properties are changed in VLCADD muscle compared to healthy muscle.

Analogous elevated changes in PCr and Pi levels during stationary exercise against a standardized submaximal workload have previously been described in patients with failing mitochondria due to a molecular defect in the respiratory chain [44,45]. Therefore, a first possible explanation would be that the mitochondrial capacity for ATP production during exercise at a workload associated with maximal rates of fat oxidation and low rates of carbohydrate utilization is lower in VLCADD muscle than in healthy muscle. Inhibition of mitochondrial oxidative ADP phosphorylation by high concentrations of incompletely oxidized long chain acyl-carnitines that may accumulate during prolonged exercise has been demonstrated *in vitro* [11]. However, we found no evidence in the kinetic measurements of post-exercise metabolic recovery that a total of 45 minutes of exercise at FATMAX workload produced any cellular condition in VLCADD muscle that interfered with mitochondrial ATP synthesis *in vivo*.

An alternative mitochondrial function-related explanation of the result could be that mitochondria in VLCADD and healthy muscle are not oxidizing the same mix of fat and carbohydrates during stationary exercise at FATMAX workload. It has been well documented ex vivo in both skeletal and cardiac muscle that changing the oxidative fuel source without changing the work (and thereby ATP turnover) rate, may cause changes in myocellular PCr and Pi levels[46,47]. If this would be the case here, it may be expected that VLCADD muscle would be oxidizing more carbohydrate, not fat, than healthy muscle. It has, however, been shown that a shift in substrate utilization towards fat, not carbohydrates, is associated with higher Pi and lower PCr levels at the same work rate [46,47]. This only leaves open the third alternative explanation that the elevated changes in quadriceps PCr and Pi concentrations in the patients were simply the result of a higher ATP turnover rate during exercise at FATMAX workload than in healthy muscle. In skeletal muscle, this metabolic flux is determined first and foremost by the myosin isoform composition of the muscle and, as such, by its fiber type composition[42]. Therefore, we investigated what change in fiber type composition of the quadriceps muscle may explain the result.

The mathematical model of oxidative ATP metabolism in human quadriceps muscle that was used to simulate oxidative ATP metabolism in human quadriceps muscle, provides a fair representation of the quantitative knowledge base on human muscle anatomy and physiology as well as the biochemistry of oxidative energy transduction. Amongst others, parameterization of the core kinetic model of myofiber oxidative ATP metabolism included model fitting to in vivo kinetic studies of ATP metabolism in human quadriceps muscle [32,48]. However, the results of the model simulations also depended on variables that we did not measure including the default fiber type composition of quadriceps muscle in our particular control group of healthy subjects, thereby introducing a degree of uncertainty in the model predictions. Therefore, the specific objective of the numerical studies was to generate rational hypotheses explaining the observed differences in quadriceps energy balance during FATMAX exercise between VLCADD patients and healthy subjects rather than specifically predict absolute values of any particular metabolic variable such as fiber type specific respiration rates during exercise at FATMAX.

A first explanation of the main result of this study proposed by the simulations was that VLCADD patients recruited a larger fraction of their total pool of SO fibers of the quadriceps than healthy subjects to maintain the workload corresponding to individual FATMAX (Table 5 and Table C in S1 File, scenario I). A corollary of this particular numerical solution is that VLCADD SO fibers produce 1.3-foldless force per contraction than in healthy controls. However, no evidence for muscle weakness has been documented in VLCADD patients [49]. Therefore, this solution was rejected. The next simplest numerical solution was that the absolute number of SO fibers in VLCADD quadriceps muscle was 30% reduced and replaced by FOG fibers (Table 5 and Table C in S1 File, scenario II). Independent data from two alternative transgenic mouse models of a long chain fatty acid oxidation defect -VLCADD knockout [50] and Steroid Receptor Coactivator-3 (SRC-3) knockout, respectively [51]- are both qualitatively and quantitatively consistent with this model prediction [50]. Specifically, in the VLCADD knockout model a slow-to-fast shift in quadriceps fiber type composition was found corresponding to 20% of the SO fibers in VLCAD knockout mice compared to wild type (WT) littermates [50]. Likewise, in mice lacking SRC-3 a trend towards an increase in fast twitch fiber type compared to WT was found which was further supported by qPCR analysis of gene markers for different fiber types[51]. Importantly, this slow-to-fast shift in skeletal muscle fiber type composition in response to either VLCADD or SRC-3 gene ablation was found without any employment of exercise or nutritional intervention [50], suggesting the adaptation may be generic to an impaired muscular capacity for long chain fatty acid oxidation[51]. In this light, it is of interest to note that in the present study, the magnitude of the change in PCr and Pi concentrations in exercising quadriceps muscle was near-identical in all five patients giving rise to the model-based prediction of a uniform change in fiber type composition of the quadriceps musle in all patients. Due to the limited number of patients that were enrolled in the investigation, however, it cannot be ruled out that this uniformity was serendipitous. Yet, it was remarkable considering the highly non-uniform clinical manifestation of the VLCADD across patients, ranging from an asymptomatic adult phenotype to severe myopathic phenotypes with elevated basal CK levels.

To our knowledge, the fiber type composition of skeletal muscle in human VLCADD has only been reported in a single case study [12]. In that particular patient, a fast-to-slow shift in fiber type composition was found [12]. However, two additional findings in that particular case report raise doubts about a strict monogenic (i.e., VLCADD) origin of the clinical phenotype of this particular patient. Firstly, it was reported that the amount of exercise required to trigger a rhabdomyolytic attack in this patient decreased with age [12]. Such a progressive time course of the disease has not been observed in any of the 45 VLCADD patients seen in our clinic; however, others have observed progressive disease with lower amounts of exercise inducing rhabdomyolysis as patients with VLCAD age (unpublished observations). Secondly, an abnormal, alkaline pH in resting muscle was found in this VLCADD case report (pH 7.30; [12]). This striking abnormality was not found in any of the five patient studied here and, in fact, more consistent with previous findings in patients with dystrophinopathies [52–54]. Muscular type I fiber predominance is likewise a common finding in dystrophin myopathies [55–57]. Therefore, we concluded that this single case report of a SO fiber type predominance in human VLCADD provided insufficient ground to reject our model-based hypothesis of a slow-to-fast fiber type transformation. Future studies of the fiber type composition of skeletal muscle in VLCADD patients are needed to rigorously test the hypothesis.

The result of this study fit the longstanding hypothesis of an energetic cause of post-exercise rhabdomyolysis in metabolic myopathies including VLCADD. Healthy active muscle derives the majority of its substrate for oxidative ATP production during exercise from intramyocellular lipid and glycogen stores [58]. Absence of any significant capability for fat oxidation such as in VLCADD renders the myofibers dependent on intracellular glycogen stores to produce ATP during exercise[51]. The capacity of these stores to produce glycosyl units for oxidative glycolysis is, however, finite [59]. As such, a molecular defect of VLCADD in and by itself puts the myofibers at risk of an energy crisis during prolonged exercise. Any slow-to-fast phenotypic adaptation of VLCADD skeletal muscle would aggravate this problem due to the threefold lower energetic contractile efficiency in combination with the twofold higher glycogenolytic capacity of FOG fibers compared to SO fibers [60]. The fact that specifically fast-twitch fibers are lost in rhabdomyolysis [61] is in support of this hypothesis.

Finally, the results of this study may provide a basis for rational therapeutic approaches to minimize risk of exertional rhabdomyolysis in myopathic VLCADD patients. Currently, no effective therapy has been developed to prevent rhabdomyolysis in VLCADD [4]. Instead, patients are typically advised to avoid any intense or prolonged exercise[17,18,62]. The long-term health outcome of lifelong restricted physical activity is, however, likewise a concern[63]. Our findings suggest that any therapeutic approach resorting in glycogen sparing during exercise should be considered for clinical testing. For one, exercise training programs to reverse any slow-to-fast phenotypic adaptation of skeletal muscle in VLCADD patients may be considered. However, such a training program should be carefully monitored and tailored to individual phenotypic traits. Secondly, novel strategies to supply VLCADD patients with alternative oxidative fuel sources during physical work should be pursued. In this light, the recent breakthrough synthesis of an ingestible ketone ester as a vehicle for establishing acute nutritional ketosis in human subjects to provide muscles with ketones as oxidative fuel source is especially promising [64]. In vivo ^31^P MRS offers a suitable, non-invasive platform to both map such personalized phenotypic traits in individual patients as well as monitor the efficacy of personalized exercise training programs.

## Supporting Information

S1 File Fiber-type-specific submodel parameterization (Table`A). Results of 3-day diary prior to second test. Mean ± SEM’s are reported **(Table B)**. Time constants of PCr recovery for various fiber type compositions of quadriceps muscle **(Table C)**. Timecourse of phosphocreatine (PCr; arbitrary units (AU)) level in quadriceps muscle of patient ID#01 immediately following exercise. The error bars show the variance in the data from AMARES fitting of the MR spectra (see Methods section). The solid red line shows the fit of a monoexponential function to the data; the dashed blue lines shows the 95% confidence interval of the fit **(Fig A)**.
(DOCX)
