# Supplementary material for: Altered Energetics of Exercise Explain Risk of Rhabdomyolysis in Very Long-Chain Acyl-CoA Dehydrogenase Deficiency
Source: PLoS One. 2016 Feb 16;11(2):e0147818. doi: 10.1371/journal.pone.0147818 (PMC4755596; doi:10.1371/journal.pone.0147818)
Supplement: S1 File — Fiber-type-specific submodel parameterization (Table`A). Results of 3-day diary prior to second test. Mean ± SEM’s are reported (Table B). Time constants of PCr recovery for various fiber type compositions of quadriceps muscle (Table C). Timecourse of phosphocreatine (PCr; arbitrary units (AU)) level in quadriceps muscle of patient ID#01 immediately following exercise. The error bars show the variance in the data from AMARES fitting of the MR spectra (see Methods section). The solid red line shows the fit of a monoexponential function to the data; the dashed blue lines shows the 95% confidence interval of the fit (Fig A). (DOCX) [file pone.0147818.s001.docx]

**Supplementary File**

**Altered energetics of exercise explain risk of rhabdomyolysis in Very Long-chain Acyl-CoA Dehydrogenase deficiency**

E.F. Diekman^1,2^, G. Visser^1,2^, J.P.J. Schmitz^3,4^, R.A.J. Nievelstein^5^, M. de Sain-van der Velden ^1^_,_ M. Wardrop^6^, W.L. Van der Pol^7^, S.M. Houten^2^, N.A.W. van Riel^4^, T. Takken^6^, J.A.L. Jeneson^6,8 #^*

**A. Supplemental Tables**

**S1 Table.** Fiber-type-specific submodel parameterization.

| **Parameter** | **Type I** | **Type IIA** | **Type IIX** | **References** |
| --- | --- | --- | --- | --- |
| Mitochondrial volume (%) | 6.0 | 4.5 | 2.3 | 1 |
| [PCr]_resting state | 0.9 x {Measured average [PCr]} | 1.1 x {Measured average [PCr]} | 1.1 x {Measured average [PCr]} | 2, 3 |
| Total creatine poolsize (mM) | [PCr]_resting state_Type I /0.85 | [PCr]_resting state_Type IIA /0.85 | [PCr]_resting state_Type IIX /0.85 | 4 |
| [Pi]_resting state (mM) | 4 | 2 | 2 | 5 |

Supplemental references:

1. Howald H, Hoppeler H Claassen H, Mathieu O Straub R. Influences of endurance training on the ultrastructural composition of the different muscle fibers in humans. Pflugers Archiv 403: 369-376, 1985.

2. Sahlin K, Soderlund K, Tonkonogi M, Hirakoba K. Phosphocreatine content in single fibers of human muscle after sustained submaximal exercise. Am J Physiol 273: C172-178, 1997.

3. Soderlund K, Hultman E. ATP and phosphocreatine changes in single human fibers after intense electrical stimulation. Am J Physiol 261: E737-741, 1991.

4. Boska M. ATP production rates as a function of force level in the human gastrocnemius/soleus using ^31P^ MRS. Magn Reson Med 32: 1-10, 1994.

5. Bottinelli R, Reggiani C. Human skeletal muscle fibers: molecular and functional diversity. Prog Biophys Mol Biol 73: 195-262, 2000

**S2 Table.** Results of 3-day diary prior to second test. Mean ± SEM’s are reported.

| **CPET test (2nd test)** |  |  |  |
| --- | --- | --- | --- |
| t=0 | **controls** | **patients** | *p-value* |
|  |  |  |  |
| Energy intake (kcal) | 2131 | 2320 | 0.55 |
| Protein intake (gram/energy%) | 88.6 (16.7) | 79.2 (14.0) | 0.31 (0.22) |
| Carbohydrate intake (gram/energy%) | 237.0 (44.5) | 294.9 (50.0) | 0.55 (0.22) |
| Total fat intake (gram/energy%) | 74.6 (32.0) | 76.1 (35.2) | 0.69 (0.42) |
| LCT intake (gram/% of total fat intake) | 74.6 (100.0) | 74.6 (85.2) | 0.69 (0.31) |
| MCT intake (gram/% of total fat intake) | 0.0 (0.0) | 8.3 (14.8) | 0.31 (0.31) |
|  |  |  |  |

**S3 Table.** Time constants of PCr recovery for various fiber type compositions of quadriceps muscle.

| case | SO fiber poolsize (% of total) | FOG fiber poolsize (% of total) | FG fiber poolsize (% of total) | time constant (s) |
| --- | --- | --- | --- | --- |
| I | 50 | 35 | 15 | 14 |
| II | 35 | 50 | 15 | 17 |

**B. Supplemental Figure**

**S1 Figure**. Timecourse of phosphocreatine (PCr; arbitrary units (AU)) level in quadriceps muscle of patient ID#01 immediately following exercise. The error bars show the variance in the data from AMARES fitting of the MR spectra (see Methods section). The solid red line shows the fit of a monoexponential function to the data; the dashed blue lines shows the 95% confidence interval of the fit.
